# Residential cognitive–behavioral therapy versus therapeutic community for patients with methamphetamine use disorders in the Philippines: A randomized controlled trial

**DOI:** 10.1016/j.abrep.2025.100605

**Published:** 2025-04-13

**Authors:** Takayuki Harada, Tomohiro Shirasaka, Toshiaki Baba, Aya Mizusawa, Alfonso Villaroman, Rosalina Noguera-Caoile, Ma. Alodia Mercado, Jasmin Peralta, Keigo Hatto, Shogo Kanamori

**Affiliations:** a College of Psychology, School of Human Sciences, University of Tsukuba, Tsukuba, Japan; b Department of Psychiatry, Teine Keijinkai Medical Center, Sapporo, Japan; c National Center for Global Health and Medicine, Tokyo, Japan; d Project for Introducing Evidence-based Relapse Prevention Programs to Drug Dependence Treatment and Rehabilitation Centers, Manila, Philippines; e Drug Abuse Treatment and Rehabilitation Center Bicutan, Taguig, Philippines; f Drug Abuse Treatment and Rehabilitation Center Dagupan, Pangasinan, Philippines; g A-Max Psychological Services, Manila, Cavite, Philippines; h Drug Abuse Treatment and Rehabilitation Center, Cebu City, Mandaue, Philippines; i Department of Community and Global Health, Graduate School of Medicine, The University of Tokyo, Tokyo, Japan

**Keywords:** Cognitive–behavioral therapy, Methamphetamine, Relapse prevention, Residential treatment, The Philippines, Therapeutic community, Well-being

## Abstract

**Introduction:**

In the Philippines, an estimated 1.8 million people use methamphetamine. Despite the government’s anti-drug campaign, repressive methods have been ineffective. Thus, treatment and human services are needed. We developed a cognitive–behavioral therapy (CBT) program for residential and rehabilitation center-based treatment and examined its effectiveness**.**

**Methods:**

In this block-randomized single-blinded trial, control group participants received therapeutic community-type treatment only, while the intervention group additionally participated in our CBT program for 6 months. Primary outcomes were methamphetamine re-use, measured by urine tests and self-report at 3-months post-discharge, and self-reported well-being at pre-discharge and 3-months post-discharge.

**Findings:**

Participants (n = 326) were randomized into intervention (n = 162) and control (n = 164) groups. At follow-up, 3/27 (11.11 %) and 1/27 (3.70 %) participants in the respective groups had positive urine tests for drug re-use (χ^2^(1) = 1.08, n.s., r = 0.037, 95 %CI [-0.064, 0.212]). The self-reported drug re-use rate was significantly higher in the intervention group (χ^2^(1) = 9.18, p < 0.01, r = 0.14 (95 %CI [0.050, 0.231]). However, pre-discharge self-rated health (F(1, 311) = 6.32, p < 0.025, η^2^ = 0.02, 95 %CI [0.001, 0.06]) and problem-focused coping (F(1, 311) = 6.50, p < 0.025, η^2^ = 0.021, 95 %CI [0.001, 0.06]) were significantly better among intervention group participants than among control participants.

**Conclusions:**

Although without significant effect on drug re-use, the intervention had a small positive effect on patients’ well-being, coping skills, and other psychological variables. Additional research is required to develop effective treatments for methamphetamine use in this region.

## Highlights

• We evaluated cognitive–behavioral therapy for methamphetamine use in the Philippines (84 characters).
• This study was conducted in a real-world setting in residential treatment facilities (84 characters).
• Cognitive–behavioral therapy improved patients’ well-being and coping skills (76 characters).
• Our findings may lead to improved efforts to provide evidence-based treatment (77 characters).
• More research is needed for effective treatment of people who use methamphetamine (81 characters).

## Graphical abstract

**Figure:**
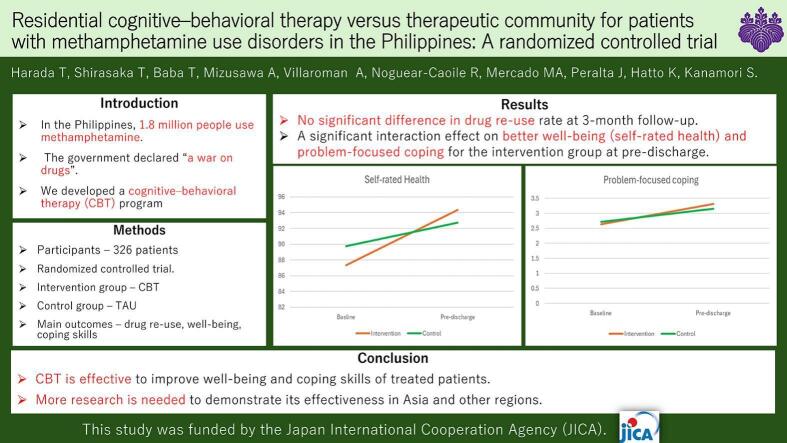

## 1 Introduction

Methamphetamine is a highly addictive substance, and its long-term use has various mental and physical effects, including anxiety, insomnia, mood disorders, cognitive dysfunction, hallucinations, and delusions. According to the World Drug Report 2023, 36 million people used amphetamines worldwide (United Nations Office on Drugs and Crime, 2023).

Methamphetamine use is a severe public health issue in the Philippines. An estimated 1.8 million people used methamphetamine in 2015 (Cousins, 2016). In 2016, former President Rodrigo Duterte declared a “war on drugs,” launched a nationwide anti-drug campaign, and implemented extrajudicial measures. Consequently, more than 1 million people who use drugs (PWUDs) surrendered to the authorities within 6 months, leading to the saturation of treatment and rehabilitation centers (TRCs) across the country (Cousins, 2016), highlighting the knowledge and capacity gaps in existing TRCs.

In response to the increased treatment demand, the Philippine Department of Health (DOH) started the Project for Introducing Evidence-Based Relapse Prevention Programs to Drug Dependence Treatment and Rehabilitation Centers (IntERlaPP), with financial and technical support from the Japan International Cooperation Agency in 2017. The DOH operates 20 TRCs nationwide and provides residential treatment for relatively severe PWUD cases. Of those, 13 facilities accommodate only males. PWUDs admitted to TRCs are required to stay in the treatment program for at least 6 months. The treatment platform is based on the therapeutic community (TC) modality, in which PWUDs live together and create their own interactive community for recovery through meetings and the fulfillment of roles and responsibilities (De Leon, 1995, Malivert et al., 2012). Patients discharged from TRCs are mandated to attend the aftercare program for 18 months.

Cognitive–behavioral therapy (CBT) has been demonstrated to be effective in the treatment of substance use disorders, including alcohol, opioid, and cocaine use (Beck, 2020, Carroll and Kiluk, 2017). However, clear evidence of its effectiveness for methamphetamine use is lacking. A meta-analysis comparing CBT for methamphetamine with other treatments found that the low-quality evidence could not substantiate its effectiveness (Harada et al., 2019). Tran et al. (2021) conducted a meta-analysis of 11 psychosocial interventions for methamphetamine use. They found that, although CBT alone was ineffective, combining multiple treatment types may effectively reduce the number of days of methamphetamine use.

The Matrix Model is a CBT-based treatment specifically designed to treat methamphetamine use (Rawson et al., 1995, Shoptaw et al., 1994). We have developed a comprehensive treatment program, named the Intensive Treatment and Rehabilitation Program for Residential Treatment and Rehabilitation Centers for Drug Dependents (INTREPRET) (Harada et al., 2021), based on the Matrix Model with consideration of Philippine cultural and social factors. INTREPRET comprises five modules: CBT, CBT-Review, Psycho-Education for Patients and Family Members, Social Support, and Self-Help Group Meeting. Herein, we conducted a randomized controlled trial (RCT) to evaluate the effectiveness of INTREPRET.

## 2 Methods

The published study protocol (Harada et al., 2021) was developed in accordance with the SPIRIT guidelines (Calvert et al., 2018, Chan et al., 2013) and the CONSORT statement (Begg et al., 1996). The study was registered in multiple clinical trial registries (number: JPRN-UMIN000038597). No significant methodological changes were made during the study.

### 2.1 Study design

This block RCT used a block size of 20. The study was approved by the Ethics Review Boards of the University of Tsukuba, Japan (T2019-70) and the Philippine DoH (SJRB-2019-27).

### 2.2 Participants

Three TRCs served as study sites, selected as representative regarding patient and facility characteristics to ensure external validity. Participant eligibility criteria were being male, age ≥ 18 years, and having used methamphetamine. Participants were recruited immediately after admission to study sites. Exclusion criteria were incapacity to participate in group sessions, inability to communicate in Tagalog, having criminal records other than illegal drug possession or use, or the presence of severe medical conditions. To avoid contamination, the intervention and control group participants were assigned residence in separate dormitories in one of the three TRCs, while the small facility sizes of the remaining two TRCs made such an arrangement impossible.

Before study participation, a study summary and a consent form were provided to all potential participants. Those who provided written consent were enrolled and assured that they could withdraw at any stage without any negative consequences.

We asked participants to cooperate in the post-test even if they dropped out so that the intention-to-treat analysis could be performed to the greatest extent possible. The collected data contained no personal information and were managed using participant ID numbers only.

### 2.3 Randomization and masking

Participants were assigned to either group using a computer-generated randomization table. One of the research collaborators created the table in advance and stored it on an office PC. The survey team in charge of recruitment and allocation was contacted by phone each time an eligible participant entered the TRCs and received instructions on group assignment. Thus, the allocation sequence was appropriately concealed from the survey team members.

Because participants knew whether they were in the intervention or control group, masking of participants was impossible. However, the outcome measurement was based solely on the participant’s ID, and the survey team members responsible for data collection and assessment did not know to which group the participant belonged. Thus, the masking of those assessing the outcomes was performed appropriately.

### 2.4 Procedures

This study evaluated the effectiveness of our INTREPRET program when integrated into an existing TC-based platform. INTREPRET mainly focuses on the following: (1) identifying triggers for drug use, (2) learning coping skills for triggers, (3) learning alternative behaviors, and (4) learning coping skills for cravings and negative emotions.

INTREPRET content was adapted through repeated discussions with local experts and DOH officials to ensure that it could be implemented in a residential setting and in the Philippine cultural context and to ensure that the DOH had a sense of ownership over the program. Subsequently, the program was translated into Tagalog, and the translation was checked multiple times by local experts. The control group participated in the existing TC-based program as scheduled by their respective TRCs. The content primarily involved group meetings, exercises, spiritual discussions, and encounter groups.

Therapists assigned to the intervention group received 5 days of training before the study. The training program included basic psychotherapy skills, instructions on conducting INTREPRET, and clinical skills in CBT and motivational interviewing. The training was delivered by local experts who had attended supervisor training on the Matrix Model. Additionally, a 3-month dry-run was conducted, during which the trainers supervised the therapists. A checklist was used to assess the fidelity and integrity of program implementation.

### 2.5 Outcomes

The primary outcomes were methamphetamine re-use, as determined based on urine tests and self-reports obtained within 3-months post-discharge, and participants’ well-being, as measured by questionnaires at three different time-points. The following questionnaires were used to assess well-being: The World Health Organization-Five Well-Being Index (WHO-5 Well-Being Index), which evaluates overall well-being (Topp et al., 2015), and the Five-Level EQ-5D (EQ-5D-5L), which evaluates health-related quality of life (Whynes & TOMBOLA Group, 2008).

The secondary outcomes were eight psychological variables (measured using questionnaires), perception of care, and aftercare program attendance rates. The questionnaires used were the following (Table 1): Drug Abuse Screening Test-20 (DAST20) (Harada et al., 2023), the Addiction Severity Index–Self Report (ASI-SR) (Cacciola et al., 2008), the Stimulant Relapse Risk Scale (SRRS) (Harada et al., 2023), the Visual Analogue Scale (VAS) for craving (Aitken, 1969), the Alcohol-Use Disorder Identification Test (AUDIT-C) (Saunders et al., 1993), the Coping Behaviors Inventory–Drug (CBI-Drug) (Litman et al., 1983), the Brief Coping Orientation to Problems Experienced (Brief COPE) (Sica et al., 1997), the Beck Depression Inventory-II (BDI-II) (Beck et al., 1996), and the Perceptions of Care (PoC) surveys (Eisen et al., 2002).

**Table 1:** Data collection tools used at different time points.

Variables/scales	Time Points			Statistical Tests Applied
	Baseline	Pre-discharge	Follow-up	
*Primary Outcomes*				
1. Urine test			X	χ^2^ test
2. Self-report drug use	X		X	χ^2^ test
3. WHO-5 Wellbeing Index	X	X	X	Repeated-measures ANOVA
4. Five-level EQ-5D (EQ-5D-5L)	X	X	X	Repeated-measures ANOVA
*Secondary Outcomes*				
5. Drug Abuse Screening Test 20 (DAST- 20)	X		X	Repeated-measures ANOVA
6. Addiction Severity Index-Self Report (ASI-SR)	X		X	Repeated-measures ANOVA
7. Stimulant Relapse Risk Scale (SRRS)	X	X	X	Repeated-measures ANOVA
8. Visual Analogue Scale (VAS) for craving	X	X	X	Repeated-measures ANOVA
9. Alcohol Use Disorder Identification Test (AUDIT-C)	X		X	Repeated-measures ANOVA
10. Coping Behaviors Inventory-Drug (CBI-Drug)	X		X	Repeated-measures ANOVA
11. Brief Coping Orientation to Problems Experienced (Brief COPE)	X	X	X	Repeated-measures ANOVA
12. Beck Depression Inventory-II (BDI-II)	X	X	X	Repeated-measures ANOVA
13. Perception of Care (PoC) Survey		X		*t*-test
14. Aftercare programme attendance			X	χ^2^ test

Note: ANOVA = Analysis of Variance.

We used the Tagalog versions of the DAST20 and SRRS, which were validated previously (Harada et al., 2023). The other eight questionnaires were translated into Tagalog by a professional translator and were tested for clarity by the patients and staff members at two TRCs.

Data were collected at the following time-points: (1) baseline, (2) pre-discharge, and (3) 3-month follow-up. The follow-up data were collected as follows: (1) self-administered questionnaires and urine tests at TRCs when participants attended the aftercare program, (2) self-administered questionnaires and urine tests at a clinic near the participants’ residences, and (3) interview-based questionnaires telephonically. Those who participated in the follow-up survey at a clinic near their residence were given 500 Philippine pesos. Similarly, those interviewed by phone were given 200 Philippine pesos.

Data collection began in February 2020 but was suspended in March 2020 due to the COVID-19 pandemic. It resumed in May 2022, and only data collected after suspension were analyzed.

### 2.6 Statistical analysis

A sample size calculation was performed with a significance level of 0.05, a statistical power of 0.95, a two-tailed test, and an estimated effect size of r = 0.23, based on similar previous RCTs, which yielded a minimum of 88 participants per group. However, as a preliminary study indicated a markedly high dropout rate (44 %), the number was conservatively set at 200 per group (400 in total). The statistical tests used for the analyses are listed in Table 1. Analyses were performed in Stata SE 17.0 (Stata Corp., College Station, TX), Mplus8 (https://www.statmodel.com/), and R 4.3.1 (https://www.r-project.org/).

## 3 Results

**Fig. 1:**
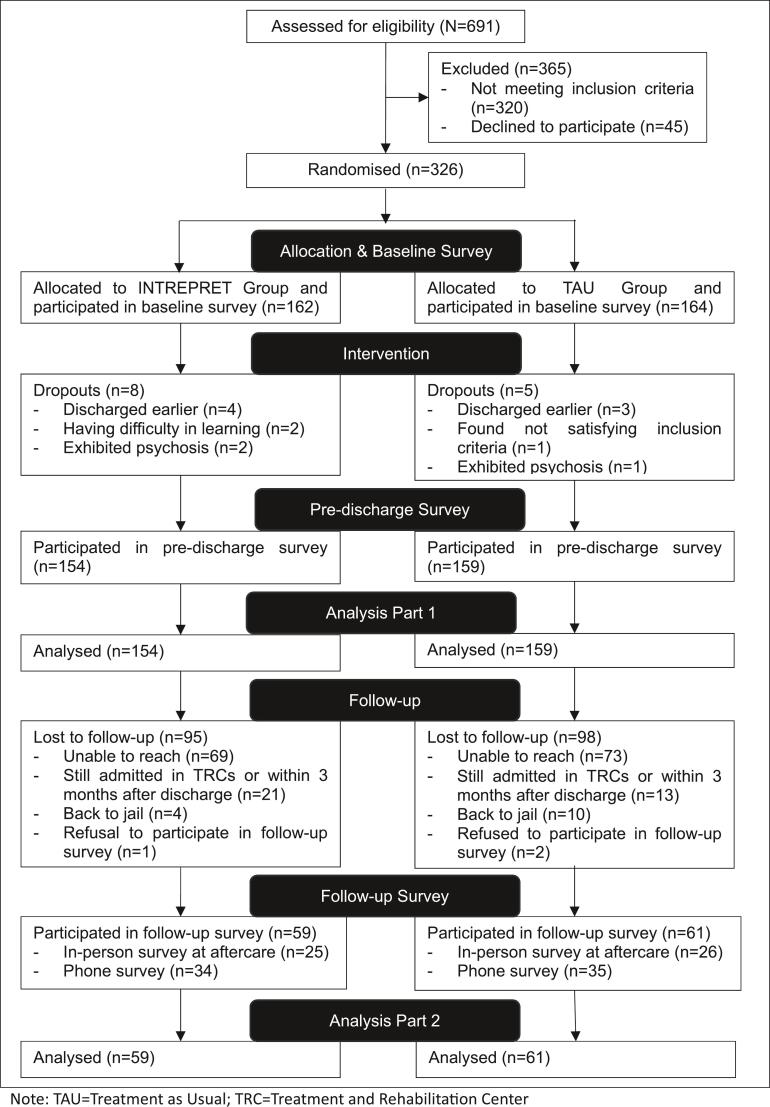
Participants flow Note: TAU = Treatment as Usual; TRC = Treatment and Rehabilitation Center.

All patients admitted to the three TRCs between May 2022 and May 2023 (N = 691) were recruited as study participants. The participant flow is shown in Fig. 1. The participants’ baseline characteristics are presented in Table 2. No harm or unexpected effects were observed.

**Table 2:** Characteristics of the study participants at the baseline stage (n = 326).

	Intervention (%) n = 162	Control (%) n = 164	Total (%) n = 326	
1. Facility				
TRC 1	90 (55.6)	92 (56.1)	182 (55.8)	
TRC 2	22 (13.6)	22 (13.4)	44 (13.5)	
TRC 3	50 (30.9)	50 (30.5)	100 (30.7)	
2. Age				
18–19	1 (0.6)	2 (1.2)	3 (0.9)	
20–24	18 (11.1)	18 (11.0)	36 (11.1)	
25–29	28 (17.3)	32 (19.6)	60 (18.5)	
30–34	33 (20.4)	34 (20.9)	67 (20.6)	
35–39	26 (16.1)	36 (22.1)	62 (19.1)	
40–44	28 (17.3)	28 (17.2)	56 (17.2)	
45–49	14 (8.6)	4 (2.5)	18 (5.5)	
50–	14 (8.6)	9 (5.5)	23 (7.1)	
3. Education				
No education	1 (0.6)	3 (1.8)	4 (1.2)	
Did not complete elementary	13 (8.0)	12 (7.3)	25 (7.7)	
Elementary graduate	10 (6.2)	15 (9.2)	25 (7.7)	
Did not complete high school	44 (27.2)	43 (26.2)	87 (26.7)	
High school graduate	72 (44.4)	78 (47.6)	150 (46.0)	
Diploma course graduate	14 (8.6)	7 (4.3)	21 (6.4)	
College graduate or higher	8 (4.9)	6 (3.7)	14 (4.3)	
4. Marital status				
Never married and single	87 (53.7)	71 (43.3)	158 (48.5)	
Married	26 (16.1)	36 (22.0)	62 (19.0)	
Living together but not married	40 (24.7)	44 (26.8)	84 (25.8)	
Divorced/Separated	8 (4.9)	11 (6.7)	19 (5.8)	
Widowed	1 (0.6)	2 (1.2)	3 (0.9)	
5. Employment Status				
Employed	33 (20.4)	44 (26.8)	77 (23.6)	
Self-employed	62 (38.3)	73 (44.5)	135 (41.4)	
Househusband	7 (4.3)	9 (5.5)	16 (4.9)	
Student	5 (3.1)	1 (0.6)	6 (1.8)	
None of the above and unemployed	55 (34.0)	37 (22.6)	92 (28.2)	
6. Frequency of methamphetamine use during the last 12 months before being admitted to TRC				
Daily	17 (10.8)	14 (8.9)	31 (9.8)	
2–5 times a week	37 (23.4)	30 (19.0)	67 (21.2)	
Weekly	36 (22.8)	31 (19.6)	67 (21.2)	
2–3 times a month	21 (13.3)	28 (17.7)	49 (15.5)	
Monthly	19 (12.0)	23 (14.6)	42 (13.3)	
Less than once a month	28 (17.7)	32 (20.3)	60 (19.0)	
7. Main route of methamphetamine administration during the last 12 months before being admitted to TRC				
Injection	4 (2.5)	3 (1.8)	7 (2.2)	
Snorting/Inhaling	153 (94.4)	150 (92.0)	303 (93.2)	
Oral (eating or drinking)	2 (1.2)	5 (3.1)	7 (2.2)	
Others	3 (1.9)	5 (3.1)	8 (2.5)	

Note: TRC = Treatment and Rehabilitation Center.

### 3.1 Primary outcome: Methamphetamine re-use rate

The primary outcome of this study was the incidence rate of methamphetamine re-use at the 3-month follow-up (Table 3). The urine test results were similar between the groups (χ^2^(1) = 1.08, n.s., 95 % confidence interval [CI] [−0.064, 0.212]). Based on self-reports, the incidence rate was significantly higher in the intervention than in the control group (χ^2^(1) = 9.18, p < 0.01, r = 0.14; 95 %CI [0.050, 0.231]).

**Table 3:** Incidence rates and the incidence rate difference are measured by urine tests and self-reports.

	Intervention			Control			Incidence Rate Difference	95 % CI	χ^2^
	Yes	No	Incidence Rate	Yes	No	Incidence Rate			
Positive in urine test (n = 54)	3	24	0.111	1	26	0.037	0.074	−0.064 to 0.212	1.08
Self-reported drug use (n = 118)	8	49	0.140	0	**61**	0.000	0.140	0.050 to 0.231	9.18***

***p < 0.01.

Note: CI = Confidence Interval.

### 3.2 Primary outcome: Well-being

Table 4 shows the mean values and F-scores for well-being, the other primary outcome. Analysis revealed a significant interaction effect on better well-being, measured by self-rated health in the EQ-5D-5L, for the intervention group at pre-discharge (F(1, 311) = 6.32, p < 0.025, η^2^ = 0.02, 95 %CI [0.001, 0.06]). No significant interaction effect was found for other variables.

**Table 4:** Mean Values and Repeated Measures ANOVA Test Results for WHO-5 Well-being and EQ-5D-5L.

	Mean (SD)						*F*-score			
	Baseline		Pre-discharge		Follow-up		Baseline & Pre-discharge		Baseline & Follow-up	
	Inter-vention n = 162	Control n = 164	Inter-vention n = 154	Control n = 159	Inter-vention n = 59	Control n = 61	Time Points	Inter-action	Time Points	Inter-action
*WHO-5 Well-being – Ratings between 0 and 100*										
1. WHO-5 Well-being Score	61.53 (21.10)	62.49 (22.94)	72.44 (19.70)	71.19 (20.19)	73.49 (18.67)	76.85 (16.81)	42.97***	0.31	25.02***	0.05
*EQ-5D-5L*										
2. QOL Score – between 0 and 1	0.917 (0.139)	0.936 (0.101)	0.928 (0.121)	0.936 (0.101)	0.961 (0.124)	0.971 (0.058)	0.73	0.43	12.99***	0.13
3. Self-rated Health – between 0 and 100	87.73 (13.55)	89.77 (12.89)	94.34 (7.86)	92.57 (8.44)	90.20 (13.25)	93.97 (8.15)	39.47***	6.32**	3.99*	0.31

*p < 0.050, **p < 0.025, ***p < 0.010.

Note: ANOVA = Analysis of Variance; EQ-5D-5L = Five-level EQ-5D.

Significant main effects of the time-points were found in the WHO-5 Well-Being Index score at pre-discharge (F(1, 309) = 42.97, p < 0.01, η^2^ = 0.12, 95 %CI [0.06, 0.19]) and follow-up (F(1, 118) = 25.02, p < 0.01, η^2^ = 0.17, 95 %CI [0.07, 0.29]), QOL score of the EQ-5D-5L at follow-up (F(1, 117) = 12.99, p < 0.01, η^2^ = 0.10, 95 %CI [0.02, 0.21]), and self-rated health of the EQ-5D-5L at pre-discharge (F(1, 311) = 39.47, p < 0.01, η^2^ = 0.11, 95 %CI [0.05, 0.18]) and follow-up (F(1, 118) = 3.99, p < 0.01, η^2^ = 0.03, 95 %CI [missing, 0.12]).

### 3.3 Secondary outcomes: Psychological scales

Table 5 shows the mean values and F-scores of the psychological scales at the three different time-points. A significant interaction on smaller scores of the ASI-SR-Drugs (F(1, 118) = 7.50, p < 0.01, η^2^ = 0.06, 95 %CI [0.005, 0.16]) was found in the intervention group at follow-up, lower scores of SRRS–compulsivity for drugs (F(1, 118) = 5.23, p < 0.025, η^2^ = 0.042, 95 %CI [0.0001, 0.13]) in the intervention group at follow-up, and higher scores of the Brief COPE–problem-focused coping (F(1, 311) = 6.50, p < 0.025, η^2^ = 0.021, 95 %CI [0.001, 0.06]) in the intervention group at pre-discharge. No other significant interaction effects were found.

**Table 5:** Mean values and repeated measures ANOVA test results for psychometric scales.

	Mean (SD)						*F*-score			
	Baseline		Pre-discharge		Follow-up		Baseline & Pre-discharge		Baseline & Follow-up	
	Inter-vention n = 162	Control n = 164	Inter-vention n = 154	Control n = 159	Inter-vention n = 59	Control n = 61	Time Points	Inter-action	Time Points	Inter-action
*DAST20 – 20 items for yes and no answers*										
1. DAST-20	6.562 (3.833)	6.488 (3.470)	−	−	5.508 (3.334)	4.951 (3.138)	−	−	3.73	0.38
*ASI-SR − 11 items with composite scores between 0 and 1*										
2. Alcohol	0.136 (0.131)	0.123 (0.138)	−	−	0.070 (0.090)	0.056 (0.075)	−	−	33.67***	0.01
3. Drugs	0.136 (0.109)	0.099 (0.089)	−	−	0.026 (0.047)	0.018 (0.032)	−	−	111.81***	7.50***
*SRRS – 35 items with ratings between 1 and 3*										
4. Anxiety and Intention to Use Drugs (AI) – 8 items	1.429 (0.376)	1.385 (0.342)	1.287 (0.323)	1.266 (0.286)	1.155 (0.218)	1.119 (0.197)	33.58***	0.08	44.76***	1.11
5. Emotionality Problems (PM) – 8 items	1.636 (0.436)	1.646 (0.460)	1.524 (0.418)	1.517 (0.363)	1.273 (0.295)	1.287 (0.319)	16.53***	0.20	54.81***	0.34
6. Compulsivity for Drugs (CD) – 4 items	1.326 (0.486)	1.244 (0.393)	1.122 (0.283)	1.138 (0.315)	1.008 (0.046)	1.041 (0.259)	34.83***	2.87	27.54***	5.23**
7. Positive Expectancies and Lack of Control Over Drug (PL) – 6 items	1.421 (0.502)	1.367 (0.435)	1.264 (0.423)	1.266 (0.39)	1.071 (0.230)	1.063 (0.159)	14.63***	0.25	42.79***	0.64
8. Lack of Negative Expectancy for the Drug (NE) – 4 items	2.028 (0.495)	2.056 (0.485)	1.731 (0.500)	1.849 (0.565)	1.949 (0.489)	1.930 (0.552)	46.43***	0.89	3.24	0.09
9. Insight into Illness (Il) – 5 items	1.579 (0.526)	1.554 (0.534)	1.371 (0.394)	1.407 (0.493)	1.220 (0.317)	1.289 (0.448)	25.02***	1.22	23.66***	3.34
10. Average of 6 subscales	1.568 (0.303)	1.539 (0.263)	1.385 (0.256)	1.407 (0.261)	1.291 (0.129)	1.288 (0.171)	68.05***	1.22	72.77***	2.37
*VAS – Ratings between 0 and 10*										
11. Current state of craving for drugs	0.874 (1.646)	0.656 (1.220)	0.436 (0.846)	0.454 (0.981)	0.217 (0.515)	0.143 (0.395)	11.39***	1.32	18.46***	1.48
12. The strongest craving for drugs in the past two weeks	0.854 (1.690)	0.652 (1.278)	0.659 (1.465)	0.404 (0.812)	0.363 (1.165)	0.144 (0.419)	4.85	0.07	12.43***	0.23
*AUDIT-C – Ratings between 0 and 20*										
13. AUDIT-C Score	2.856 (2.826)	2.506 (2.800)	−	−	2.169 (2.422)	2.311 (2.618)	−	−	6.33**	0.01
*CBI-Drug – 36 items with ratings between 0 and 3*										
14. CBI Average Score	1.399 (0.514)	1.321 (0.520)	−	−	1.317 (0.367)	1.377 (0.407)	−	−	3.80	1.48
*Brief COPE – 28 items with ratings between 1 and 4*										
15. All items – 28 items	2.285 (0.587)	2.325 (0.566)	2.625 (0.407)	2.530 (0.419)	2.343 (0.364)	2.494 (0.386)	56.05***	4.72	0.93	1.78
16. Problem-Focused Coping – 8 items	2.637 (0.835)	2.709 (0.812)	3.313 (0.604)	3.156 (0.630)	2.979 (0.579)	3.125 (0.551)	122.50***	6.60**	10.80***	0.10
17. Emotion-Focused Coping – 12 items	2.278 (0.628)	2.305 (0.638)	2.595 (0.486)	2.493 (0.473)	2.285 (0.385)	2.425 (0.393)	37.56***	3.30	0.02	2.29
18. Avoidant Coping – 8 items	1.944 (0.535)	1.971 (0.516)	1.982 (0.417)	1.959 (0.433)	1.794 (0.426)	1.967 (0.484)	0.06	0.87	1.69	3.04
*BDI-II – Ratings between 0 and 63*										
19. BDI-II Score	9.646 (9.276)	9.220 (9.832)	7.862 (8.223)	7.417 (7.733)	3.034 (5.034)	2.475 (3.650)	13.26***	0.02	74.84***	0.02

**p < 0.025, ***p < 0.010.

Note: ANOVA = Analysis of Variance; DAST20 = Drug Abuse Screening Test 20; ASI-SR = Addiction Severity Index-Self Report; SRRS = Stimulant Relapse Risk Scale; VAS = Visual Analogue Scale for craving; AUDIT-C = Alcohol Use Disorder Identification Test; CBI-Drug = Coping Behaviors Inventory-Drug; Brief COPE = Brief Coping Orientation to Problems Experienced; BDI-II = Beck Depression Inventory II.

The analysis also revealed significant main effects of the time-points on better scores on the two ASI-SR scales at follow-up, seven SRRS scales at pre-discharge, six SRRS scales at follow-up, two VAS scales at both pre-discharge and follow-up, the AUDIT-C at follow-up, three Brief COPE scales at pre-discharge, one Brief COPE scale at follow-up, and the BDI-II at both pre-discharge and follow-up.

### 3.4 Secondary outcomes: Perception of care survey

The PoC survey was administered to participants pre-discharge (n = 313). No significant difference in the mean factor scores was found between the groups (Table 6).

**Table 6:** Perception of care survey results at the pre-discharge stage (n = 313).

	Mean Factor Score (SD)		t
	Intervention n = 154	Control n = 159	
1. Information Received – 3 items	95.67 (16.92)	94.65 (16.81)	0.53
2. Interpersonal Aspects of Care – 7 items	83.37 (23.79)	79.87 (25.19)	1.26
3. Continuity/ Coordination of Care – 5 items	87.89 (16.88)	86.99 (18.03)	0.45
4. Global Evaluation of Care – 3 items	89.52 (18.92)	86.22 (21.04)	1.46
5. All items – 18 items	87.61 (15.78)	85.28 (17.17)	1.25

### 3.5 Secondary outcome: Attendance rate for aftercare programs

The average numbers of days on which participants attended the aftercare during the follow-up were 4.42 ± 4.96 days (n = 53) for the intervention and 4.86 ± 5.46 days (n = 62) for the control group. The between-group difference was −0.44 days, which was not statistically significant (t(113) = 0.45, 95 %CI [-0.51, 2.38], n.s.).

### 3.6 Additional statistical analysis

The results included many missing data points. A mechanism of missing not-at-random (MNAR) could be plausible, where the probability of missing depends on the observed and missing data. When the probability of missing is not random (i.e., missing completely at random), coping strategies, such as listwise or pairwise deletion, can distort the results. Therefore, we performed an additional analysis to account for missing data.

Even for MNAR mechanisms, the missing at random assumption can be satisfied by conditioning on variables related to missing data. Therefore, we used the inclusive-analysis strategy for the urine test and self-reported drug use to identify auxiliary variables and performed a probit regression with the intervention dummy variable and the auxiliary variables as independent variables. Regression analysis was performed with the baseline values as control variables, in addition to the intervention dummy and auxiliary variables for the other variables for which data were available at two time-points (DAST20, ASI-SR, CBI, and AUDIT-C). For the auxiliary variables, we identified indicators associated with the two outcomes and their respective missing indicators from baseline variables. Specifically, variables with a standardized mean difference of ± 0.20 or more between the missing and non-deficient groups for each of the two outcomes, or with a partial correlation of ± 0.30 or more between the two outcomes conditioned by the intervention, were employed as auxiliary variables. The robust maximum likelihood method was used to estimate the probit regression and tested using Wald’s test. The intervention was a significant positive predictor only when self-reported drug use was the dependent variable (β = 0.80, SE = 0.07, p < 0.001). Thus, again, the intervention group was more likely to report drug use than was the control group (Table 7).

**Table 7:** Results of regression analysis using auxiliary variables and probit regression.

Dependent variable	*β*	*SE*	*z*	*p*	Auxiliary variable
DAST20	0.16	0.17	0.94	0.35	Age, education, frequency of amphetamine use, CBI
ASI-SR (Drug)	0.07	0.10	0.74	0.46	Age, education, frequency of amphetamine use, CBI, DAST20
ASI-SR(Alcohol)	0.01	0.09	1.13	0.26	Age, education, frequency of amphetamine use, CBI, DAST20
CBI	−0.10	0.09	−1.16	0.25	Age, education, frequency of amphetamine use, DAST20
AUDIT-C	−0.02	0.09	−0.18	0.86	Age, education, frequency of amphetamine use, CBI, DAST20, ASI-SR(Alcohol)
	Probit regression				
Urine test	−0.05	2.44	−0.02	0.99	Age, education, marital status, frequency of amphetamine use, WHO-5, QOL, EQ, DAST20, ASI-SR, SRRS, VAS, CBI-Drug, Brief COPE
Self-reported drug use	0.80	0.07	11.36	< 0.01	Age education, frequency of amphetamine use, EQ, DAST20, ASI-SR(Drug), SRRS, CBI

Note: DAST20 = Drug Abuse Screening Test 20; ASI-SR = Addiction Severity Index-Self Report; SRRS = Stimulant Relapse Risk Scale; VAS = Visual Analogue Scale for craving; CBI-Drug = Coping Behaviors Inventory-Drug; Brief COPE = Brief Coping Orientation to Problems Experienced.

**Fig. 2:**
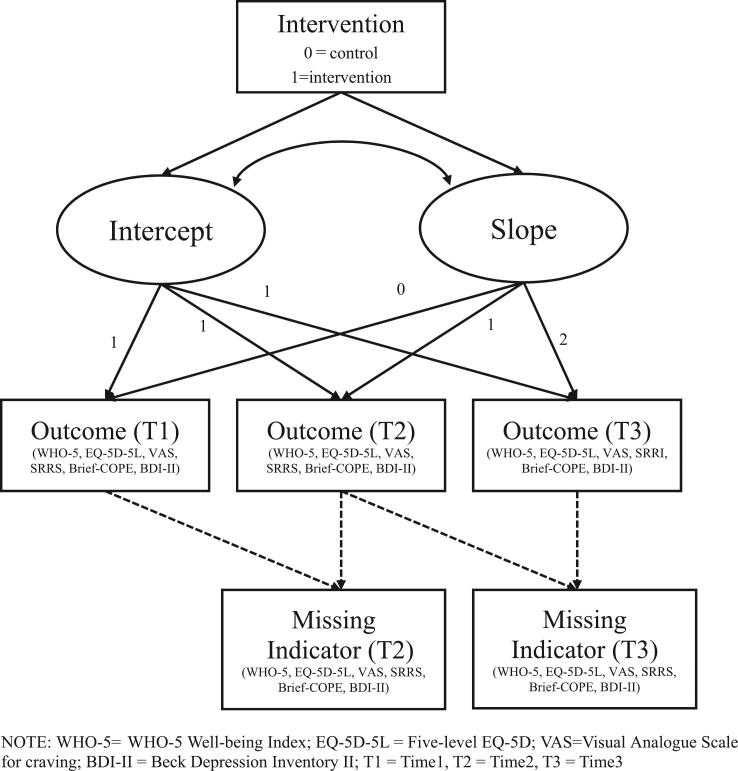
Diagram of the Diggle-Kenward model. NOTE: WHO-5 = WHO-5 Well-being Index; EQ-5D-5L = Five-level EQ-5D; VAS = Visual Analogue Scale for craving; BDI-II = Beck Depression Inventory II; T1 = Time1, T2 = Time2, T3 = Time3.

For variables with data at three time-points (WHO-5, EQ, VAS for craving, SRRS, COPE, BDI), analyses were conducted using the Diggle–Kenward model, which applies a latent growth model, where the missing indicator at one time-point is set to relate to the observed data at the previous time-point and the missing data at the same time-point. Analysis showed that none of the interventions significantly predicted the slope (Table 8, Fig. 2).

**Table 8:** Growth curve estimates from Diggle-Kenward model.

	WHO-5			EQ-5D-5L			VAS			SRRS			Brief-COPE			BDI-II		
Effect	Est.		*SE*	Est.		*SE*	Est.		*SE*	Est.		*SE*	Est.		*SE*	Est.		*SE*
Intercept (mean)	61.79	^**^	0.17	88.49	^**^	0.96	0.77	^**^	0.10	0.24	^**^	0.03	2.30	^**^	0.04	10.44	^**^	0.73
Slope (mean)	11.50	^**^	0.10	7.10	^**^	0.77	−0.58	^**^	0.07	0.96	^**^	0.04	0.29	^**^	0.04	−6.08	^**^	0.47
Intercept (variance)	163.81	*	0.09	81.31	^**^	13.19	0.98	^**^	0.14	0.01	*	0.00	0.16	*	0.03	47.66	^**^	7.19
Slope(variance)	49.91		28.67	43.61	^**^	8.40	0.34	^**^	0.07	0.11	^**^	0.04	0.08	^**^	0.02	7.50	^**^	2.07
Intervention → Intercept	−0.12		2.35	−0.89		1.36	0.17		0.14	0.01		0.04	0.00		0.06	0.48		1.04
Intervention → Slope	−0.03		1.95	0.57		1.08	−0.10		0.10	0.00		0.05	0.02		0.05	−0.19		0.66

*p < 0.05, ^**^ p < 0.01.

NOTE: WHO-5 = WHO-5 Well-being Index; EQ-5D-5L = The Five-Level EQ-5D; SRRS = Stimulant Relapse Risk Scale; VAS = Visual Analogue Scale for craving; CBI-Drug=Coping Behaviors Inventory-Drug; Brief COPE = Brief Coping Orientation to Problems Experienced; BDI-II = Beck Depression Inventory II.

## 4 Discussion

In this study, we developed a residential CBT-based treatment program (INTREPRET) for methamphetamine-use disorder in the Philippines and evaluated its effectiveness, to establish whether INTREPRET, when integrated into existing TC-based treatments, would be more effective than TC treatment alone.

The primary outcomes were methamphetamine re-use at 3-months post-discharge as well as participants’ well-being. No difference was found in urine test results according to treatment type. However, a significantly greater proportion of INTREPRET participants reported drug re-use. Several explanations are possible. First, INTREPRET could have been insufficiently effective in preventing drug re-use. If so, the program needs to be revised after collecting information on related studies and opinions of local experts and patients, to improve its effectiveness. Second, although paradoxical, it remains possible that, because of certain effects of INTREPRET, more INTREPRET participants affirmed drug re-use in the self-report. The traditional TC takes a zero-tolerance position on drug use and teaches that drug re-use is unacceptable and should be avoided at all costs (De Leon, 1995, Vanderplasschen et al., 2013). Additionally, PWUDs may be subject to punishment in the Philippines. Therefore, individuals may be strongly resistant to honestly reporting drug re-use. On the other hand, INTREPRET distinguishes between lapse and relapse in line with the relapse prevention concept and emphasizes that, while lapse is not desirable, it is common during the recovery process and that persons in recovery should honestly acknowledge it and learn from the experience to avoid further relapse (Marlatt & Donovan, 2005). These differences in therapeutic attitudes toward lapse may manifest as differences in the attitudes of INTREPRET participants, making them more likely to report lapses honestly.

Third, as often noted for CBT, the effects could have occurred with a delay (Rawson et al., 2002). CBT focuses on skill learning, and it may take some time to practice the skills learned during treatment and implement them effectively in real life. Moreover, because the participants were receiving treatment in a residential facility, they could not use these skills in real life until after leaving the facility. Therefore, they may not yet have exercised sufficient skills to cut off drug temptation and cope with cravings. It is assumed that multiple factors, such as those described above, acted together to produce this result.

The other primary outcome was participants’ well-being. Recently, addiction treatment goals have shifted away from abstinence to the improvement of the well-being of PWUDs (United Nations Office on Drugs and Crime, 2019). Additionally, questions about the effectiveness of residential and coercive treatment and concerns about human rights are increasing (Lunze et al., 2018). Overall, participants’ well-being improved significantly over time. In particular, the self-rated health of the INTREPRET participants improved significantly at pre-discharge as compared to that of the control participants. This may reflect the essential attitude of INTREPRET, which values the importance of a patient’s life and health over zero tolerance. Moreover, therapists had the utmost respect for the patient’s human rights, rather than teaching them only to quit drugs. Therapists supported them in achieving a healthy life without drugs and in taking care of their mental and physical health. In a qualitative study conducted simultaneously by the same authors (Kanamori et al., 2024), INTREPRET participants’ narratives also mentioned that they had a better relationship with therapists and were treated with more respect than they had been in TC-type treatments in the past. These treatment values may have influenced patients’ self-rated health.

The secondary outcomes, addiction severity, relapse risk, and coping skills improved significantly over time in both groups, suggesting the effectiveness of residential treatment, regardless of the treatment type used. Additionally, the intervention group also had significantly decreased addiction severity and compulsivity for drug use than did the control group at follow-up, and increased problem-focused coping at pre-discharge. This may be due, in large part, to the focus of INTREPRET on training coping skills for dealing with craving.

Thus, although INTREPRET had limited effects on drug use, it significantly affected participants’ well-being, reduced relapse risk, and improved coping skills. Our study results are valuable in that they can play a significant role in changing the treatment climate and values. They can be expected to contribute to improved efforts to address drug use as a public health issue in the Philippines and to respecting the human rights and well-being of PWUDs. This change in therapeutic attitude was clearly emphasized in the United Nations General Assembly Special Session on the World Drug Problem (United Nations Office on Drugs and Crime, 2016). This should be a pillar of future drug-addiction treatment. In the Philippines and other Southeast Asian countries, where human rights violations against PWUDs have been a significant concern (Lunze et al., 2018), this shift in values is significant.

This study had some limitations, most of which are related to this being a real-world trial, rather than one conducted under strict laboratory-like conditions. First, the dropout rate at follow-up was considerable, which reduced statistical power. Although certain criteria were applied to the participants, almost all patients included in the study had relatively severe conditions, and their motivation for treatment, cognitive ability, and other characteristics varied, which affected dropout. Second, the therapists were not necessarily proficient in CBT-based treatments. To compensate for this, multiple pre-trial training rounds were conducted to ensure treatment fidelity and integrity. However, this approach is not always adequate. Third, the follow-up period was short. If, as noted earlier, at least 6 months are needed for skills to be fully mastered, a short follow-up period may have resulted in outcomes being collected too early, which may have impacted the observed drug re-use rates in this real-world setting. Fourth, this study included adult male patients only, which may affect the generalizability of the results.

However, very few trials have compared CBT with other types of treatment for methamphetamine use disorder, particularly in a real-world setting (Harada et al., 2019). In this sense, our finding of a specific effect of TC, as a control treatment, and demonstration that CBT may be able to increase this effect, is valuable. Furthermore, this study is valuable because few RCTs have evaluated the treatment of methamphetamine use, and even fewer studies have been conducted outside Western countries. To the best of our knowledge, no such studies have been conducted in the Philippines. The findings of this study have valuable implications for the future development of treatment of drug-use disorders in non-Western countries, with due consideration for human rights.

## CRediT authorship contribution statement

**Takayuki Harada:** Writing – review & editing, Writing – original draft, Supervision, Project administration, Methodology, Investigation, Funding acquisition, Formal analysis, Data curation, Conceptualization. **Tomohiro Shirasaka:** Writing – review & editing, Writing – original draft, Project administration, Methodology, Investigation, Conceptualization. **Toshiaki Baba:** Methodology, Investigation, Conceptualization. **Aya Mizusawa:** Writing – review & editing, Writing – original draft, Project administration, Investigation, Conceptualization. **Alfonso Villaroman:** Project administration. **Rosalina Noguera-Caoile:** Project administration. **Ma. Alodia Mercado:** Project administration. **Jasmin Peralta:** Conceptualization. **Keigo Hatto:** Writing – review & editing, Writing – original draft, Formal analysis. **Shogo Kanamori:** Writing – review & editing, Writing – original draft, Project administration, Methodology, Investigation, Formal analysis, Data curation, Conceptualization.

## Funding

Japan International Cooperation Agency. The funder had no role in the study design, data collection and analysis, decision to publish, or preparation of the manuscript.

## Declaration of competing interest

The authors declare that they have no known competing financial interests or personal relationships that could have appeared to influence the work reported in this paper.

## Data Availability

I have shared the link to my data
