# Supplementary material for: Residential cognitive–behavioral therapy versus therapeutic community for patients with methamphetamine use disorders in the Philippines: A randomized controlled trial
Source: Addict Behav Rep. 2025 Apr 13;21:100605. doi: 10.1016/j.abrep.2025.100605 (PMC12032182; doi:10.1016/j.abrep.2025.100605)
Supplement: Supplementary Data 1 [file mmc1.pdf]

Harada et al. Trials (2021) 22:909
https://doi.org/10.1186/s13063-021-05882-6

Trials

STUDY PROTOCOL Open Access

Evaluation of the Intensive Treatment and Rehabilitation Program for Residential Treatment and Rehabilitation Centers (INTREPRET) in the Philippines: a study protocol for a randomized controlled trial

Takayuki Harada[1*], Toshiaki Baba[2], Tomohiro Shirasaka[3] and Shogo Kanamori[4]

Abstract

Background: The Philippines has been severely affected by the methamphetamine crisis. The government has launched a policy war against drug use, although the severe sanctions imposed on drug users have been criticized internationally. To help implement a more effective and humane approach to drug use, this study aimed to introduce a comprehensive treatment program for methamphetamine users based on cognitive-behavioral therapy (CBT) whose effectiveness will be evaluated through a randomized controlled trial.

Methods: Methamphetamine users admitted into government-run rehabilitation facilities are recruited and randomly assigned to either a CBT-based treatment program or existing therapeutic community (TC)-based treatment. The CBT treatment program was developed based on the Matrix Model that considers cultural and social factors in the Philippines. After 6 months of treatment, there will be a three-month follow-up, when the participants' drug use (tested through urine testing) and other psychological variables, including craving, coping skills, and well-being, will be compared. Potential participants are given a summary of the study and a consent form. The consent form is signed and dated by participants prior to their study participation. Ethical approval was obtained prior to the commencement of the study.

Discussion: This is the first randomized controlled trial to compare the residential CBT program and the TC model for methamphetamine users in the Philippines. The study aims to fill the current knowledge and capacity gaps by introducing a CBT-based treatment program to improve the psychosocial well-being of drug users in the Philippines. Moreover, if the effectiveness of the treatment program is demonstrated, anti-drug campaigns and severe sanctions against drug users may be reconsidered.

Trial registration: UMIN Clinical Trials Registry JPRN-UMIN000038597. Registered on 15 November 2019. Protocol version October 17, 2021 ver.2

Keywords: Methampetamine use disorders, Cognitive-behavioral therapy, The Philippines, Residential treatment, Therapeutic community, Matrix Model

* Correspondence: tkharada77@gmail.com
[1]Faculty of Human Sciences, University of Tsukuba, 3-29-1 Otsuka, Bunkyo-ku, Tokyo 112-0012, Japan
Full list of author information is available at the end of the article

© The Author(s). 2021 **Open Access** This article is licensed under a Creative Commons Attribution 4.0 International License, which permits use, sharing, adaptation, distribution and reproduction in any medium or format, as long as you give appropriate credit to the original author(s) and the source, provide a link to the Creative Commons licence, and indicate if changes were made. The images or other third party material in this article are included in the article's Creative Commons licence, unless indicated otherwise in a credit line to the material. If material is not included in the article's Creative Commons licence and your intended use is not permitted by statutory regulation or exceeds the permitted use, you will need to obtain permission directly from the copyright holder. To view a copy of this licence, visit http://creativecommons.org/licenses/by/4.0/. The Creative Commons Public Domain Dedication waiver (http://creativecommons.org/publicdomain/zero/1.0/) applies to the data made available in this article, unless otherwise stated in a credit line to the data.

Background

Introduction

The crisis in methamphetamine use has spread worldwide in recent times, particularly in Southeast Asia, with methamphetamine seizures in the region reaching 1000 tons in 2018, which was five times larger than that in 2013 [1]. The Philippines is one of the most severely affected countries in this region, where drug use has become a major public health concern. National statistics indicated a prevalence rate of illegal drug use in 2.3% of the population in 2015 or the equivalent of 1.8 million people in the 10–69 years age range [2].

Under these circumstances, President Rodrigo Duterte declared a war on drugs and launched a national anti-drug campaign in 2016, involving dismantling clandestine methamphetamine laboratories and arresting drug suppliers [3]. Over one million drug users made themselves known to the authorities and sought treatment and social support [4]. This unprecedented demand for drug use treatment services highlighted knowledge and capacity gaps in existing services. Therefore, there is an urgent need to develop evidence-based treatment that considers the country's cultural and social background. It is estimated that 1% of those who came forward were high-risk users [5], who usually have multiple and intensive treatment needs.

The Philippine Department of Health is responsible for providing residential treatment for high-risk drug users and operates 12 treatment and rehabilitation centers (TRCs) nationwide. They rely heavily on a therapeutic community (TC) model, which is very popular. However, no robust evaluation has been undertaken of this model, and there is wide variability in practice. Furthermore, one meta-analysis did not identify any significant benefits for the rehabilitation of drug users using that model [6]. Additionally, the TC model employed in the TRCs varies. Its major elements include group meetings, religious gatherings, physical exercise, and housekeeping activities. These elements are usually delivered for 6 months in an unstructured manner and their effectiveness remains unevaluated.

Cognitive-behavioral thearpy (CBT) is commonly used to treat individuals with drug problems worldwide and is popular in Western countries. However, there is no clear evidence for its effectiveness in treating methamphetamine use disorders in terms of high-quality research [7]. CBT addresses multiple treatment needs of drug users, including building skills to resist drug use, replacing drug-using activities with constructive and rewarding activities, improving problem-solving skills, and facilitating better interpersonal relationships in a structured manner [8, 9].

The Matrix Model is a specifically designed CBT-based treatment model to treat stimulant users and has been evaluated many times in several countries, such as the USA [10–12] as well as Asian countries, including Japan [13] and Iran [14]. We developed a comprehensive CBT treatment program based on the Matrix Model considering Philippine cultural and social factors, to treat methamphetamine users in TRCs, namely, an "Intensive Treatment and Rehabilitation Program for Residential Treatment and Rehabilitation Centers" (INTREPRET).

Aims and objectives

This study aimed to fill knowledge and capacity gaps through introducing INTREPRET into the Philippines and comparing it with current TC-based treatment, through conducting a parallel group randomized controlled trial to evaluate its effectiveness concerning subsequent drug use and psychosocial well-being. The primary objectives of this study are:

1. To establish an evidence-based residential treatment model for national dissemination. The findings of the study are expected to bring long-term benefits to high-risk drug users and communities alike through determining whether this treatment model works in the Philippine residential setting or needs further improvement before it is scaled up nationwide.
2. To direct policymakers towards the introduction of more effective treatment, to contribute to improved treatment services for drug users, and to facilitate approaches more respectful of drug users' human rights and dignity.

Methods

Overview

The trial design is a parallel group randomized control trial. The study protocol was developed by the authors (chaired by the first author) following the CONSORT Statement [15] and the Standard Protocol Items: Recommendations for Interventional Trials (SPIRIT) [16]. The protocol, research method, and collected data have been checked and overseen by a Research Working Group (RWG) headed by the program manager of the Philippine Dangerous Drugs Abuse and Prevention and Treatment Program (DDAPTP). The RWG is composed of both Japanese and Philippine researchers, including the authors of this protocol, and responsible for auditing core trial processes.

Study setting

INTREPRET will be introduced into three Department of Health-operated TRCs, selected as representative in terms of patient and facility characteristics, to ensure external validity.

Participant enrolment and allocation

The participants are recruited from among newly admitted patients in three TRCs starting from April 2022; the process will continue for approximately three years until the total number of participants from those TRCs reaches 400. We are in close contact with the staff in charge of recruiting to monitor the enrolment process.

Those who meet the eligibility criteria are selected as participants for the study. After obtaining informed consent, participants are randomly assigned to either the intervention group or the control group. During the intervention phase, the patients in the intervention group will receive INTREPRET, while those in the control group will receive current TC-based treatment or treatment as usual (TAU).

Randomization of eligible patients is performed by independent research assistants who are not part of the TRC administration. When a new eligible patient is admitted to the TRCs, the staff inform the allocation team, and the patient is randomly assigned to either of the groups using a pre-determined computer-generated table. The table has a block size of 20 with ten intervention and ten control statuses listed in a random sequence. A new table is generated and used in every 20 newly admitted patients. The tables are placed in a sealed envelope and retained by the data collection team in locked storage until the end of the trial.

The blinding of participants in terms of interventions they receive cannot be ensured because of the nature of the intervention. However, research assistants responsible for recruiting participants, and data collection and analysis are adequately blinded.

Eligibility criteria

The following inclusion criteria apply:

1) Residential patients newly admitted to one of the three pilot TRCs
2) Being male
3) Being 18 years of age or older
4) Ever having used methamphetamine

The following exclusion criteria apply:

1) Those not capable of participating in group sessions
2) Those who cannot communicate in Tagalog
3) Those with criminal records other than for possession of illegal drugs, possession of drug paraphernalia, or use of illegal drugs
4) Those with severe medical conditions

Participation is limited to males because the overwhelming majority of drug users are male, so their clinical needs have a high priority. The patients in the intervention group are separated from those in the control group (and those not participating in the study) by placing them in a designated dormitory to avoid possible between-group communication. They are also instructed not to disclose treatment materials to non-intervention group participants. No specific dormitories are designated for the control group, whose participants stay in non-intervention group dormitories together with those not participating in the study.

While allowing for dropout or withdrawal, data are still collected for intention-to-treat analysis for all dropout/withdrawal cases. The following dropout/withdrawal criteria apply:

1) Explicit statement of intent to reject receiving treatment
2) Explicit statement of withdrawal from study participation
3) Failure to receive treatment more than 5 times
4) Failure to comply with institutional regulations

Sample size calculation

Based on recently published findings from a systematic review that included studies with a similar design [7], this study considered a minimum detectable effect of 0.23 and a population standard deviation of 0.42. With a significance level of 0.05, and a power of 0.95, a sample size of 88 participants per group (or 176 patients in total) was determined as needed to test the causal effect of the intervention.

Based on preparatory field study experience, it is possible that more than half of the participants may drop out of the study by not showing up for the follow-up interviews in the three months after discharge. Given a coverage rate of 44%, the final required sample size needed to be adjusted to 200 patients per group (or 400 patients in total) to achieve a sample size of 88 patients per group (or 176 patients in total).

Intervention

The original Matrix Model was translated into the Tagalog language, and local researchers and practitioners checked the translation multiple times. Additionally, the contents were tailored to the cultural and social background of the Philippines and specific treatment needs of drug users in the Philippines and modified to be conducted in residential TRC settings.

INTREPRET is composed of five components: CBT sessions, CBT-review sessions, psycho-education sessions, social support sessions, and self-help group meetings. The main contents of the CBT sessions are as follows: (1) identifying triggers for drug use, (2) learning coping skills for triggers, (3) learning alternative behaviors, (4) learning coping skills for craving, (5) learning

coping skills for negative emotions (anger management, stress management), and (6) rebuilding social support networks.

To implement these five components of INTREPRET, the following materials have been developed:

1) A service provider manual [17]
2) A patient workbook
3) Slides for psycho-education sessions
4) Flipcharts for social support sessions

In implementing INTREPRET, service providers follow the protocols described in the manual [17], which elaborates the organization and resource requirements for the implementation of different treatment components and the use of specific materials and tools. It also provides operating procedures and quality standards to facilitate group sessions for each treatment component.

During the intervention, the patients in the intervention group receive INTREPRET through participating in three sessions of CBT, one session of CBT review, one session of psycho-education, two sessions of social support, and one session of self-help group meetings per week. All sessions, each lasting 60 min, are provided in groups. INTREPRET can be completed within 26 weeks or 6 months. Other than these CBT interventions, participants join other TC-based activities, such as physical activities and religious gatherings. Table 1 shows a sample timetable with INTREPRET program components incorporated into the TC-based treatment platform.

Patients in the control group receive TAU based on the TC approach. Typically, patients participate in a variety of daily activities designed by the TRCs, including therapeutic meetings, physical activities, education, religious gatherings, and house-keeping activities [18]. During the trial, the participants receive ancillary care, including medical care other than treatment for drug use and the opportunity to participate in religious services. Following discharge from the treatment program, all participants will receive aftercare services that include booster sessions and family therapy.

Therapists

The therapists in both groups are psychologists or social workers. The psychologists who provide the INTREPRET sessions to the intervention group receive specifically developed 5-day training for this study, consisting of lectures and hands-on group sessions on (1) INTREPRET administration, (2) facilitation standards, (3) CBT, and (4) motivational interviewing, followed by 3-month dry run sessions. The

Table 1 Sample timetable with INTREPRET program components incorporated into therapeutic community-based treatment platform

<table>
<tr><th>Time</th><th>Mon</th><th>Tue</th><th>Wed</th><th>Thu</th><th>Fri</th></tr>
<tr><td>05:00 - 06:00</td><td colspan="5">Rising Time/ Job Function</td></tr>
<tr><td>06:00 - 06:30</td><td colspan="5">Morning Exercise</td></tr>
<tr><td>06:30 - 08:15</td><td colspan="5">Wash-up/ Breakfast</td></tr>
<tr><td>08:15 - 08:30</td><td colspan="5">Pre-morning Meeting</td></tr>
<tr><td>08:30 - 09:30</td><td colspan="5">Morning Meeting</td></tr>
<tr><td>09:30 - 09:45</td><td colspan="5">Departmental Meeting</td></tr>
<tr><td>09:45 - 10:00</td><td colspan="5">Follow-up</td></tr>
<tr><td>10:00 - 11:00</td><td>CBT (1)</td><td>Athletics</td><td>CBT (2)</td><td>Athletics</td><td>CBT (3)</td></tr>
<tr><td>11:00 - 12:00</td><td colspan="5">Lunch</td></tr>
<tr><td>12:00 - 13:00</td><td colspan="5">Personal Time</td></tr>
<tr><td>13:00 - 14:00</td><td colspan="5">Job Functions</td></tr>
<tr><td>14:00 - 15:00</td><td>Recreation/ Vocational</td><td>SS (1)</td><td>Spiritual</td><td>SS (2)</td><td>Recreation/ Vocational</td></tr>
<tr><td>15:00 - 16:00</td><td>Encounter Group</td><td>PE</td><td>Static Group</td><td>SHGM</td><td>CBT-R</td></tr>
<tr><td>16:00 - 16:30</td><td colspan="5">Spiritual Enhancement Activity</td></tr>
<tr><td>16:30 - 19:00</td><td colspan="5">Personal Time/ Wash-up</td></tr>
<tr><td>19:00 - 20:00</td><td colspan="5">Evening Meeting</td></tr>
<tr><td>20:00 - 21:00</td><td colspan="5">Journal Writing/Responsible Interaction</td></tr>
<tr><td>21:00</td><td colspan="5">"Off the Floor"</td></tr>
</table>

CBT: cognitive behavioral therapy, PE: psycho-education, SHGM: self-help group meeting, SS: social support meeting. All these activities are treatment elements of INTREPRET

trainers are experienced psychologists and psychiatrists who had participated in Matrix training in the USA in 2017 and 2018. Moreover, treatment integrity is monitored by trainers during the dry run period using a fidelity checklist developed before the trial [17]. The researchers monitor treatment implementation on a random basis to ensure the quality of treatment using a fidelity checklist.

Data collection and research instruments

Outcome data are collected in three phases: pre-treatment, post-treatment, and 3-month follow-up (Table 2). To collect data on the primary outcome (i.e., drug use), urine testing for stimulants is to be implemented only during follow-up. In addition, self-administered questionnaires are used to collect outcome data on self-reported stimulant use and to determine patient demographic and psychosocial variables. To measure psychological variables, several psychometric scales are to be employed at baseline, pre-discharge, and follow-up, as follows:

1) A Drug Abuse Screening Test 20 (DAST 20) that screens drug-related disorders [19]
2) The Addiction Severity Index-Self Report (ASI-SR) that evaluates the severity of drug-related problems [20]
3) The Simulant Relapse Risk Scale (SRRS) that evaluates relapse risk of stimulant use [21]
4) The Visual Analogue Scale (VAS) for craving that evaluates the subjective magnitude of drug craving [22]
5) The Coping Behaviors Inventory-Drug (CBI-Drug) that evaluates coping behaviors related to drug use [23]
6) The Brief Coping Orientation to Problems Experienced (Brief COPE) that evaluates the general coping repertoire [24]
7) The World Health Organization Five Well-being Index (WHO-5 Well-being Index) that assesses overall well-being [25]
8) The Five-Level EQ-5D (EQ-5D-5L) that evaluates health-related quality of life, including mobility, self-care, usual activities, pain/discomfort, and anxiety/depression [26]
9) The Beck Depression Inventory II (BDI-II) that screens for depression [27]
10) Perceptions of care surveys (PoC) that evaluate self-reported care satisfaction [28]

The DAST 20 and the SRRS were translated into Tagalog and back-translated into English. The original and back-translated versions were checked for discrepancies by three researchers including a native Tagalog speaker, and final versions were agreed. In terms of reliability, the DAST 20 had an α value of 0.81. Concerning validity, receiver-operating-characteristic analysis, featuring diagnoses from independent doctors, returned an acceptable area-under-curve value of 0.62. Acceptable

Table 2 Assessment at different time points

	Time point		
Variables/scales	Pre-treatment	Post-treatment	3-month follow-up
Socio-demographics	X		
Drug use experience	X		
Urine test			X
Self-report drug use	X		X
DAST 20	X		X
ASI-SR	X		X
SRRS	X	X	X
VAS for craving	X	X	X
AUDIT	X		X
CBI-Drug	X		X
Brief COPE	X	X	X
WHO-5 Well-being Index	X	X	X
EQ-5D-5L	X	X	X
BDI-II	X	X	X
PoC		X	

DAST Drug Abuse Screening Test, *ASI-SR* Addiction Severity Index-Self Report, *SRRS* Stimulant Relapse Risk Scale, *VAS* Visual Analogue Scale, *AUDIT* Alcohol Use Disorder Identification Test, *CBI-Drug* Coping Behaviors Inventory-Drug, *COPE* Coping Orientation to Problems Experienced, *WHO-5 Well-being Index*, the World Health Organization Five Well-being Index, *EQ-5D-5L* Five-level EQ-5D, *BDI* Beck Depression Inventory, *PoC* Perceptions of Care Surveys

reliability was also confirmed for the SRRS, with an α value of 0.89. The correlation analysis of subjective drug craving (measured using the VAS) and the SRRS revealed a significant positive correlation (r = 0.19, p < 0.001), indicating adequate validity.

To ensure the anonymity and privacy of the participants, all data are de-identified and managed using codes at the data entry and analysis stages. Data are to be anonymized in a linkable fashion, involving a separate table that links the participants' codes and names, and carefully stored with access only by designated personnel. All data are collected by the trained data collection team, and researchers monitor the data collection activities on a random basis to ensure compliance with the protocol.

Data analysis

The following two types of regression equations are estimated to calculate the causal effect of providing residential treatment on outcomes:

1) For the primary outcome indicator of urine testing, regression model (1) was estimated to test any statistically significant difference in means between the intervention and control groups:

$$Y_{ij} = \alpha + \beta T_i + \theta X_i + \nu_j + \omega_{ij} \tag{1}$$

where Y_{ij} is the urine test result of patient i at TRC j, α is a constant that gives the value of the urine test result for the control group, T_i is the treatment dummy, X_i is the set of patient characteristics to be controlled for, such as age and education, ν_j is a TRC-level error term, and ω_{ij} is an individual error term.

This study estimates the coefficient of the treatment dummy (β), which shows the between-group difference. Since the urine test has a binary result, the log odds of the outcome are to be estimated as a linear combination of the independent variables using logistic regression (i.e., logit (Y_{ij})) rather than linear regression.

2) For the secondary outcome indicators of the psychometric tests, which are to be measured both at baseline and follow-up, regression model (2) with an interaction term is estimated to calculate a difference-in-difference (DD) estimate that relies on a comparison of the intervention and control groups before and after the intervention [29]:

$$Y_{ijt} = \alpha + \beta T_{i1} t + \rho T_{i1} + \gamma t + \theta X_i + \nu_{jt} + \omega_{ijt} \tag{2}$$

where Y_{ijt} is the psychometric test result of patient i at TRC j at time t, α is a constant giving the average value of the psychometric test result for the comparison group at time t_0 (baseline), T_{i1} is the treatment dummy, t is the time dummy, X_i is the set of patient characteristics to be controlled for, ν_j is a TRC-level error term, and ω_{ij} is an individual error term.

The study estimates the coefficient (β) on the interaction between the treatment dummy (T_{i1}) and the time dummy (t), which gives the average DD effect of the intervention. In addition to this interaction term, the variables T_{i1} and t are included separately to identify any separate mean effects of time as well as the effect of being targeted versus not being targeted.

Patient and public involvement

Psychometric tools for outcome measurement have been pretested on approximately 40 patients in two TRCs and feedback comments obtained from the patients and staff members who observed the pre-test sessions. The treatment programs were implemented prior to the commencement of the study at all sites and feedback obtained from the patients and used to develop the tools and programs and to administer the study.

Ethics and dissemination

This study does not anticipate that the participants will suffer any adverse effects, either physically or mentally. To our knowledge, previous studies with similar interventions have not reported any serious adverse effects. In cases of any significant negative events, the intervention would cease or be modified.

The following measures are implemented to ensure appropriate ethical standards:

1) Potential participants are given a summary of the study and a consent form. The consent form must be signed and dated by the participants prior to participation.
2) The participants are duly informed that they can withdraw from the study at any time without any negative consequences.
3) Participants are compensated for inconvenience and expenses when they attend the follow-up interview. Participants showing up and cooperating in the follow-up receive an honorarium of 500 pesos, whereas those responding to an interview by telephone receive 200 pesos.
4) All patient data are treated as confidential data. Any personally identifiable information is removed from

the datasets in a linkable anonymizing manner. Each participant has a unique study identification number, linked to their personal information, and the list for linkable anonymizing is kept in a locked cabinet in the project office.

5) The consent forms and the questionnaire forms will be kept in a lockable cabinet at a Department of Health office for 5 years after all data collection is collected, and then shredded and disposed of.
6) This study has been approved by the Single Joint Research Ethics Board of the Department of Health, Republic of the Philippines (SJREB-2019-27) and the University of Tsukuba Faculty of Human Sciences Ethics Committee, Japan (T2019-70).
7) No researchers have any competing interests concerning the current study.

The results of the study are intended for dissemination to a wide range of audiences through presentations and publication. The results will be reported on clinicaltrials.org.

Any modifications to the protocol that may affect the conduct of the study, potential benefits to the patient, or patient safety, including changes in study objectives, study design, patient population, sample sizes, study procedures, or significant administrative aspects, requires a formal amendment to the protocol. Any such amendment must be agreed by the RWG and approved by the relevant research ethics board and committee prior to implementation.

Discussion

This is the first randomized controlled trial to compare the residential CBT program and the TC model for methamphetamine users in the Philippines. The study aims to fill the gaps in the current knowledge and capacity by introducing a CBT-based treatment program to improve the psychosocial well-being of drug users in the Philippines.

Based on scientific evidence, many drug users in the Philippines do not have access to effective treatment, and consequently, their health and psychosocial well-being are threatened. Furthermore, for society as a whole, the drug problem presents challenges to all members' safety and well-being. The drug problem cannot be solved by punishment or violence. The United Nations suggested that it is important to provide appropriate support to drug users while promoting the protection of and respect for human rights and the dignity of all individuals in the context of drug programs, strategies, and policies [29].

If this study can introduce an effective treatment to the Philippines, thereby improving the drug users' health and well-being, it can help improve the health, safety, and well-being of individuals, families, communities, and society. It is also expected that policymakers will recognize the benefits of the treatment and support drug users' rehabilitation and social reintegration, rather than promoting punishment and exclusion.

Abbreviations

INTREPRET: Intensive Treatment and Rehabilitation Program for Residential Treatment and Rehabilitation Centers; JICA: Japan International Cooperation Agency; CBT: Cognitive-behavioral therapy; TC: Therapeutic community

Acknowledgements

We would like to express our sincere gratitude to the RWG members and the JICA project members, Ivanhoe Escartin, Madelaine Casimiro, Alfonso Villaroman, Jasmin Peralta, Joseph Fama, Trinidad Purugganan, Alodia Mercado, Aleli Sudiacal, Juan Perez IV, Jocelyn Sosito, Tomoyuki Sho, Flerida Chan, Chika Asakawa, Aya Mizusawa, Marcelyn Bonhaon, Amando Francisco Jr., Ric Bernardino, and Sabrina Tayo.

Authors' contributions

TH was involved in designing and writing the protocol and in supervising the whole process of the study. TB was involved in designing the protocol and writing the draft. TS reviewed and revised the protocol, and SK was involved in designing and writing the draft and administrating the study process. All authors contributed to the refinement of the study protocol and approved the final manuscript.

Funding

This study is part of a technical assistance project funded by the Japan International Cooperation Agency (JICA). The research cost is funded by the JICA, whereas Philippine Department of Health personnel provide both the intervention and control groups with clinical and other services as well as covering any other operating costs of the three TRCs. These funding sources have no role in the design of this study and its execution, analyses, interpretation of the data, or decision to submit results.

Availability of data and materials

The datasets generated and/or analyzed during the current study are available from the Harvard Database repository, https://dataverse.harvard.edu/dataset.xhtml?persistentId=doi:10.7910/DVN/CPRRYA
Registration number: 10.7910/DVN/CPRRYA registered on 9 January 2021.

Declarations

Ethics approval and consent to participate

This study has been approved by the Single Joint Research Ethics Board of the Department of Health, Republic of the Philippines (SJREB-2019-27) and the University of Tsukuba Faculty of Human Sciences Ethics Committee (T2019-70). Potential participants are given a summary of the study and a consent form to be signed and dated by them before participation.

Consent for publication

Not applicable

Competing interests

The authors declare that they have no competing interests.

Author details

[1]Faculty of Human Sciences, University of Tsukuba, 3-29-1 Otsuka, Bunkyo-ku, Tokyo 112-0012, Japan. [2]National Center for Global Health and Medicine, Tokyo, Japan. [3]Department of Psychiatry, Teine Keijinkai Medical Center, Sapporo, Japan. [4]Department of Community and Global Health, Graduate School of Medicine, The University of Tokyo, Tokyo, Japan.

Received: 26 April 2021 Accepted: 25 November 2021
Published online: 11 December 2021

References

1. United Nations Office on Drugs and Crime. World Drug Report, 2020. Available: https://wdr.unodc.org/wdr2019/ (Accessed 20 Jan 2020).

2. Board DD. Nationwide Survey on the Nature and Extent of Drug Abuse in the Philippines. Quezon City, Philippines; 2015.
3. Hechanova M, Alianan A, Calleja M, et al. The development of a community-based drug intervention for Filipino drug users. J Pacific Rim Psychol. 2018;12:E12. https://doi.org/10.1017/prp.2017.23.
4. Cousins S. Five thousand dead and counting: the Philippines' bloody war on drugs. BMJ. 2016;355:i6177. https://doi.org/10.1136/bmj.i6177.
5. Cepeda M. DDB wants law institutionalizing community-based drug treatment. Rappler 2016. Available: https://www.rappler.com/nation/147020-ddb-law-institutionalize-community-based-drug-rehabilitation (Accessed 20 Jan 2020).
6. Smith LA, Gates S, Foxcroft D. Therapeutic communities for substance related disorder. Cochrane Database Syst Rev. 2006;1:CD005338. https://doi.org/10.1002/14651858.CD005338.pub2.
7. Harada T, Tsutomi H, Mori R, Wilson DB. Cognitive-behavioural treatment for amphetamine -type stimulants (ATS)-use disorders. Cochrane Database Syst Rev. 2019;4:CD011315. https://doi.org/10.1002/14651858.
8. National Institute of Drug Abuse. Principles of drug addiction treatment: a research-based guide. 3rd ed; 2012. Available: https://www.drugabuse.gov/sites/default/files/podat_1.pdf
9. Marlatt GA, Witkiewitz K. Relapse prevention for alcohol and drug problems. In: Marlatt GA, Donovan DM, editors. Relapse prevention: Maintenance strategies in the treatment of addictive behaviors. 2nd ed. New York: The Guilford Press; 2005. p. 1–44.
10. Huber A, Ling W, Shoptaw S, Gulati V, Brethen P, Rawson R. Integrating treatments for methamphetamine abuse: a psychosocial perspective. J Addict Dis. 1997;16(4):41–50. https://doi.org/10.1080/10550889709511142.
11. Obert JL, McCann MJ, Marinelli-Casey P, et al. The matrix model of outpatient stimulant abuse treatment: history and description. J Psychoactive Drugs. 2000;32(2):157–64. https://doi.org/10.1080/02791072.2000.10400224.
12. Rawson RA, McCann MJ, Flammino F, et al. A comparison of contingency management and cognitive-behavioral approaches for stimulant-dependent individuals. Addiction. 2006;101(2):267–74. https://doi.org/10.1111/j.1360-0443.2006.01312.x.
13. Harada T. The randomized controlled trial of the prison-based Japanese Matrix Program (J-MAT) for methamphetamine abusers. Japanese J Alcohol Stud Drug Depend. 2012;47:298–307 doi: http://ci.nii.ac.jp/naid/10031143289/ja/.
14. Amiri Z, Beheshteh M, Mehrdad S. Evaluating the efficacy of regulated 12-session Matrix Model in reducing susceptibility in methamphetamine-dependent individuals. I J Med Res Health Sci. 2016;5:77–85.
15. Schultz KF, Altman DG, Moher D, et al. CONSORT 2010 statement: updated guidelines for reporting parallel group randomised trials. BMJ. 2010;340:c332. https://doi.org/10.1136/bmj.c332.
16. Chan A-W, Tetzlaff JM, Altman DG. SPIRIT 2013 Statement: defining standard protocol items for clinical trials. Ann Intern Med. 2016;158:200–7. https://doi.org/10.7326/0003-4819-158-3-201302050-00583.Requests.
17. Department of Health. Repulic of the Philippines. Service provider's manual: Intensive treatment and rehabilitation program for residential treatment and rehabilitation ceners forderug dependents (INTREPRET). Republic of the Philippines, Manila: 2020. Available: https://www.jica.go.jp/project/philippines/013/materials/ku57pq00003ud3mz-att/material_01.pdf
18. Gavin DR, Ross HE, Skinner HA. Diagnostic validity of the Drug Abuse Screening Test in the assessment of DSM-III drug disorders. Br J Addict. 1989;84(3):301–7. https://doi.org/10.1111/j.1360-0443.1989.tb03463.x.
19. Cacciola JS, Pecoraro A, Alterman AI. Development of ASI psychiatric severity cut-off scores to identify co-occurring psychiatric disorders. Int J Ment Health Addict. 2008;6(1):77–92. https://doi.org/10.1007/s11469-007-9103-7.
20. Ogai Y, Haraguchi A, Kondo A, Ishibashi Y, Umeno M, Kikumoto H, et al. Development and validation of the Stimulant Relapse Risk Scale for drug abusers in Japan. Drug Alcohol Depend. 2007;88(2-3):174–81. https://doi.org/10.1016/j.drugalcdep.2006.10.005.
21. Gould D, Kelly D, Goldstone L, Gammon J. Examining the validity of pressure ulcer risk assessment scales: developing and using illustrated patient simulations to collect the data. J Clin Nurs. 2001;10(5):697–706. https://doi.org/10.1046/j.1365-2702.2001.00525.x.
22. Litman GK, Stapleton J, Oppenheim AN, Peleg BM. An instrument for measuring coping behaviours in hospitalized alcoholics: implications for relapse prevention treatment. Br J Addict. 1983;78(3):269–76. https://doi.org/10.1111/j.1360-0443.1983.tb02511.x.
23. Sica C, Novara C, Dorz S, Sanavio E. Coping strategies: evidence for cross-cultural differences? A preliminary study with the Italian version of coping orientations to problems experienced (COPE). Pers Individ Dif. 1997;23(6):1025–9. https://doi.org/10.1016/S0191-8869(97)00112-8.
24. Topp CW, Østergaard SD, Søndergaard S, Bech P. The WHO-5 well-being index: a systematic review of the literature. Psychother Psychosom. 2015;84(3):167–76. https://doi.org/10.1159/000376585.
25. Whynes DK, TOMBOLA Group. Correspondence between EQ-5D health state classifications and EQ VAS scores. Health Qual Life Outcomes. 2008;6(1):1–9. https://doi.org/10.1186/1477-7525-6-94.
26. Beck AT, Steer RA, Ball R, Ranieri WF. Comparison of Beck Depression Inventories-IA and-II in psychiatric outpatients. J Pers Assess. 1996;67(3):588–97. https://doi.org/10.1207/s15327752jpa6703_13.
27. Eisen SV, Wilcox M, Idiculla T, Speredelozzi A, Dickey B. Assessing consumer perceptions of inpatient psychiatric treatment: the perceptions of care survey. Jt Comm J Qual Improv. 2002;28(9):510–26. https://doi.org/10.1016/S1070-3241(02)28056-6.
28. Khandker SR, Koolwal GB, Samad HA. Handbook on impact evaluation: Quantitative methods and practices. Washington, DC: The World Bank; 2010. https://doi.org/10.1596/978-0-8213-8028-4.
29. United Nations Office on Drugs and Crime. Outcome document of the 2016 United Nations General Assembly Special Session on the world drug problem: Our joint commitment to effectively addressing and countering the world drug problem. New York: United Nations; 2016.

Publisher's Note

Springer Nature remains neutral with regard to jurisdictional claims in published maps and institutional affiliations.

Ready to submit your research? Choose BMC and benefit from:

- fast, convenient online submission
- thorough peer review by experienced researchers in your field
- rapid publication on acceptance
- support for research data, including large and complex data types
- gold Open Access which fosters wider collaboration and increased citations
- maximum visibility for your research: over 100M website views per year

At BMC, research is always in progress.

Learn more biomedcentral.com/submissions
